# Fooling Examples: Another Intriguing Property of Neural Networks

**DOI:** 10.3390/s23146378

**Published:** 2023-07-13

**Authors:** Ming Zhang, Yongkang Chen, Cheng Qian

**Affiliations:** National Key Laboratory of Science and Technology on Information System Security, Beijing 100101, China

**Keywords:** fooling examples, neural networks, transferability, adversarial examples

## Abstract

Neural networks have been proven to be vulnerable to adversarial examples; these are examples that can be recognized by both humans and neural networks, although neural networks give incorrect predictions. As an intriguing property of neural networks, adversarial examples pose a serious threat to the secure application of neural networks. In this article, we present another intriguing property of neural networks: the fact that well-trained models believe some examples to be recognizable objects (often with high confidence), while humans cannot recognize such examples. We refer to these as “fooling examples”. Specifically, we take inspiration from the construction of adversarial examples and develop an iterative method for generating fooling examples. The experimental results show that fooling examples can not only be easily generated, with a success rate of nearly 100% in the white-box scenario, but also exhibit strong transferability across different models in the black-box scenario. Tests on the Google Cloud Vision API show that fooling examples can also be recognized by real-world computer vision systems. Our findings reveal a new cognitive deficit of neural networks, and we hope that these potential security threats will be addressed in future neural network applications.

## 1. Introduction

Neural networks are well known to achieve comparable or superior performance to humans in many challenging tasks, including image classification [1], speech recognition [2] and game playing [3]. However, a growing body of research demonstrates that neural networks also face various security threats, which include evasion [4], poisoning [5], extraction [6], and inference [7]. The threat of evasion attacks against machine learning models was first investigated by [8]. Subsequently, Szegedy et al. [9] experimentally discovered the existence of adversarial examples, which can be effectively used in evasion attacks against neural networks. Since then, adversarial examples, as an “intriguing property” of neural networks [9], have attracted increasing research attention.

Adversarial examples were originally synthesized by adding carefully crafted perturbations to benign examples. These perturbations are imperceptible to humans but can easily fool a neural network model. Subsequently, other types of adversarial examples have been presented, such as natural adversarial examples [10], semantic adversarial examples [11], and unrestricted adversarial examples [12]. The concept of adversarial examples has accordingly been generalized to refer to a category of examples that can fool neural networks but not humans.

**Figure 1:**
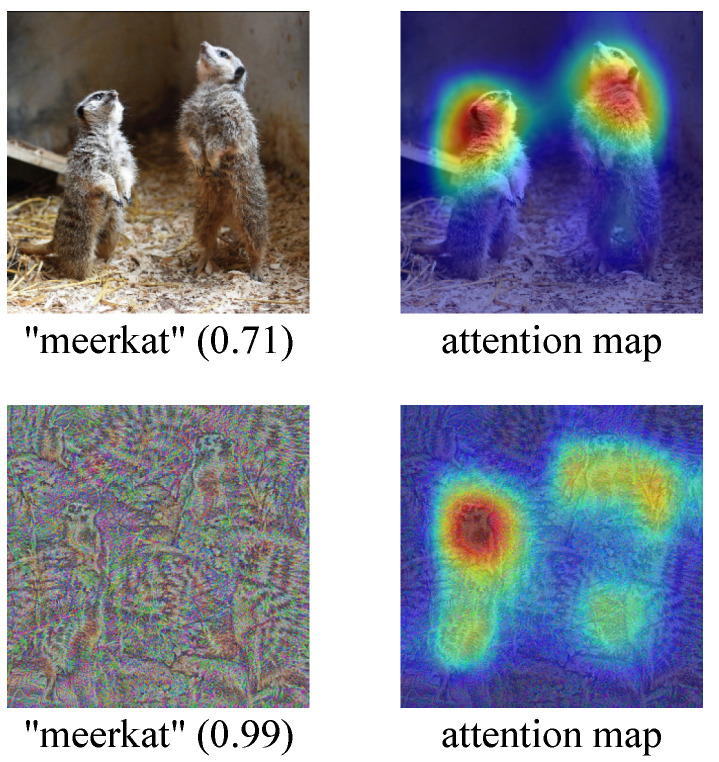
Prediction results of a natural example (**top left**) and a fooling example (**bottom left**), along with their corresponding attention maps on ResNet50 [1].

In this paper, we present another intriguing property of neural networks: the fact that well-trained models believe some examples to be recognizable objects (often with high confidence), while humans cannot recognize such examples. We refer to this category of examples, namely those that can be recognized by neural networks but cannot be recognized by humans, as “fooling examples”. As is evident, the fooling example is a new concept that differs from the adversarial example, as the latter refers to an example that can be recognized by both humans and neural networks, although neural networks give an incorrect prediction. Figure 1 shows the prediction results of a natural example and a fooling example, along with their corresponding attention maps [13,14] on ResNet-50 [1]. We can see that just like the natural example, the fooling example is also recognized as “meerkat” by the network, with even higher confidence.

The contributions of this work can be summarized as follows:

- We propose the concept of “fooling examples” and discuss the ways in which they differ from adversarial examples.
- We propose an iterative method for generating fooling examples, which combines techniques such as momentum, diverse inputs, and translation invariance to improve the transferability of fooling examples.
- We systematically evaluate the performance of our proposed method. The results indicate that fooling examples can not only be generated on white-box models, but can also be transferred to black-box models and even recognized by real-world computer vision systems.

## 2. Related Work

To the best of our knowledge, the concept of “fooling examples” is proposed for the first time in this paper; as a result, there is little existing related work in this field. Perhaps the most relevant work is the study conducted by Nguyen et al. in [15]. The authors found that deep neural networks can be easily fooled into making high-confidence predictions for unrecognizable images, which are referred to as “fooling images”. In [15], the authors mainly used two evolutionary algorithms [16] and one gradient ascent algorithm [17] to find unrecognizable examples. The gradient ascent algorithm is derived from [17], and it maximizes the softmax output for classes using gradient ascent in the image space. However, the method we proposed for generating fooling examples is an upgraded version of the iterative fast gradient sign method (I-FGSM) [18]. It combines techniques such as momentum [19], diverse inputs [20], and translation invariance [21], and can effectively improve the transferability of fooling examples.

Since our method of generating fooling examples is inspired by existing methods of generating adversarial examples [22,23,24], we also introduce some related work on adversarial examples here. Szegedy et al. [9] were the first to propose the concept of adversarial examples, and accordingly developed a box-constrained L-BFGS method for generating such examples. Goodfellow et al. proposed the fast gradient sign method (FGSM) [25] for efficiently generating adversarial examples. Kurakin et al. extended FGSM to an iterative version, i.e., I-FGSM [18]. To improve the transferability of adversarial examples, Dong et al. proposed the momentum iterative fast gradient sign method (MI-FGSM) [19]; for their part, Xie et al. proposed the diverse inputs iterative fast gradient sign method (DI^2^-FGSM) [20], after which Dong et al. proposed the translation-invariant iterative fast gradient sign method (TI^2^-FGSM) [21].

## 3. Fooling Examples vs. Adversarial Examples

We believe that the fooling examples we present in this paper are fundamentally different from the adversarial examples that have been extensively studied. In this section, we provide a formal characterization of adversarial examples and fooling examples.

Let I be the set of all input examples under consideration. We consider a neural network classifier $f:I_{t}\subseteq I\to \left\{1,2,\cdots ,k_{f}\right\}$ that can give a prediction for any example in $I_{t}$, where $I_{t}$ denotes the set of $k_{f}$ categories of natural examples that can be recognized by the classifier. Suppose $o:I\to \left\{1,2,\cdots ,k_{o}\right\}\cup \left\{unk\right\}$ is an oracle that takes an example in I and outputs one of $k_{o}$ labels, or a label unk if the example is unrecognizable. In addition, we denote the discriminator of humans as $h:I\to \left\{1,2,\cdots ,k_{h}\right\}\cup \left\{unk\right\}$; that is, the humans can recognize an example in I as a label in $\left\{1,2,\cdots ,k_{h}\right\}$ or will assign a label unk if the example is unrecognizable. We assume $k_{f}<k_{h}\leq k_{o}$. Equipped with these notations, we provide the following definitions for adversarial examples and fooling examples.

**Definition** **1** (Adversarial examples)**.** *An adversarial example is defined to be any example in $E^{adv}≜\left\{x\in I_{t}\;|\;f\left(x\right)\neq h\left(x\right)=o\left(x\right),f\left(x\right)\in \left\{1,2,\cdots ,k_{f}\right\},h\left(x\right)=o\left(x\right)\in \left\{1,2,\cdots ,k_{f}\right\}\right\}$.*

**Figure 2:**
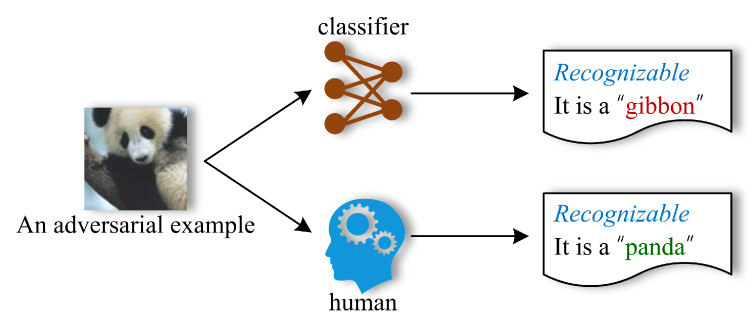
The recognization results of an adversarial example by the classifier and the human.

In other words, the adversarial example can be recognized by both humans and the classifier; however, the classifier gives an incorrect prediction. Figure 2 illustrates the recognization results of an adversarial example by the classifier and the human.

**Definition** **2** (Fooling examples)**.** *A fooling example is defined to be any example in $E^{fool}≜\left\{x\in I\;|\;f\left(x\right)\neq h\left(x\right)=o\left(x\right),f\left(x\right)\in \left\{1,2,\cdots ,k_{f}\right\},h\left(x\right)=o\left(x\right)=unk\right\}$.*

**Figure 3:**
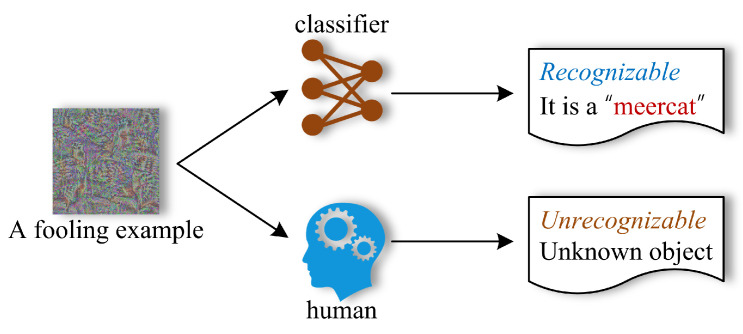
The recognization results of a fooling example by the classifier and the human.

In other words, the fooling example cannot be recognized by humans; however, the classifier believes it to be a recognizable object (even with high confidence). Figure 3 illustrates the recognization results of a fooling example by the classifier and the human.

## 4. Methodology

Fooling examples are defined as examples that can be recognized by neural networks, but cannot be recognized by humans. Theoretically, the generation of fooling examples requires human intervention, which may be a process of human–machine cooperation. However, human involvement will further complicate the process of generating fooling examples. In fact, the generation of fooling examples can be accomplished in a relatively simple way, which is described in detail in this section.

### 4.1. Problem Specification

**Figure 4:**
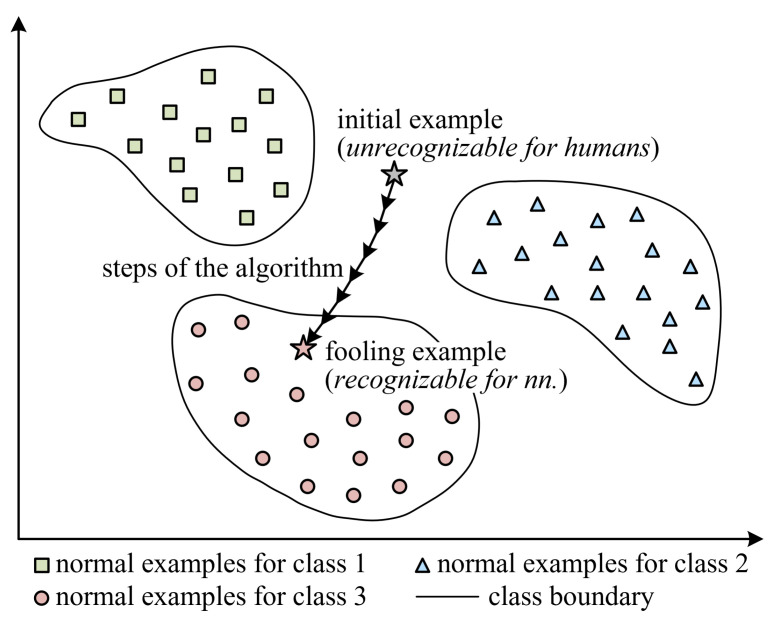
Illustration of generating a fooling example. (1) Finding an initial example that is unrecognizable for humans; (2) making minor changes to the initial example using a specific algorithm and obtaining a fooling example that is recognizable for neural networks.

As illustrated in Figure 4, the process of generating a fooling example can be divided into two phases: (a) finding an initial example that is unrecognizable for humans (e.g., random noise, all-white or all-black images); (b) making minor changes to the initial example using a specific algorithm and obtaining a fooling example that is recognizable for neural networks. Because the fooling example has a very small distinction from the initial example, it is unrecognizable for humans, but is recognizable for neural networks.

Next, we present a formalized description of the process of fooling example generation. Let $x^{init}$ denote the initial example and $x^{fool}$ denote the generated fooling example. Given a classifier *f*, we want the fooling example $x^{fool}$ to be recognized as a label *y*, i.e., $f(x^{fool})=y$. If the adversary’s loss function of the classifier is denoted as *J*, the process of generating the fooling example $x^{fool}$ can be formally expressed as a matter of solving the following constrained optimization problem:

(1) $$\underset{x^{fool}}{\arg\min}J\left(x^{fool},y\right);\;s.t.\;{∥x^{fool}-x^{init}∥}_{\infty }\leq \epsilon$$

where ϵ is the maximum threshold of the $L_{\infty }$-norm distance (i.e., ${∥\cdot ∥}_{\infty }$) between $x^{fool}$ and $x^{init}$, and guarantees that $x^{fool}$ has a very small distinction from $x^{init}$; thus, $x^{fool}$ is still unrecognizable for humans.

Solving the optimization problem (1) requires calculating the gradient of the loss function with respect to the input. Since the problem (1) is similar to the problem of generating targeted adversarial examples, the classical methods for generating adversarial examples, such as I-FGSM [18], MI-FGSM [19], DI^2^-FGSM [20], and TI^2^-FGSM [21], can be extended for generating fooling examples.

### 4.2. Gradient-Based Iterative Methods

In this paper, we consider four basic gradient-based iterative methods for generating fooling examples.

**Iterative Fast Gradient Sign Method for Generating Fooling Examples (I-FGSM^fool^).** I-FGSM [18] can be extended into a method for generating fooling examples, which is denoted as I-FGSM^fool^ and can be expressed as follows:

(2) $$\begin{array}{cc} \; & x_{0}^{fool}=x^{init}\\ \; & x_{t+1}^{fool}={\mathrm{Clip}}_{x}^{\epsilon }\left\{x_{t}^{fool}-\alpha \cdot \mathrm{sign}\left(\nabla_{x}J\left(x_{t}^{fool},y\right)\right)\right\} \end{array}$$

where $x_{0}^{fool}=x^{init}$ means that $x_{0}^{fool}$ is initialized with an example that is unrecognizable for humans (e.g., random-noise, all-white or all-black images); $x_{t}^{fool}$ denotes the intermediate example at the *t*-th iteration; ${\mathrm{Clip}}_{x}^{\epsilon }\left\{\cdot \right\}$ indicates that the example generated in each iteration is clipped within the ϵ-ball of the initial example $x^{init}$; finally, α is the step size at each iteration.

**Momentum Iterative Fast Gradient Sign Method for Generating Fooling Examples (MI-FGSM^fool^).** Similarly, MI-FGSM [19] can be extended into a method for generating fooling examples, which is termed as MI-FGSM^fool^ and can be expressed as follows:

(3) $$\begin{array}{cc} \; & x_{0}^{fool}=x^{init}\\ \; & g_{t+1}=\mu \cdot g_{t}+{\frac{\nabla_{x}J\left(x_{t}^{fool},y\right)}{∥\nabla_{x}J\left(x_{t}^{fool},y\right)∥}}_{1}\\ \; & x_{t+1}^{fool}={\mathrm{Clip}}_{x,\epsilon }\left\{x_{t}^{fool}-\alpha \cdot \mathrm{sign}\left(g_{t+1}\right)\right\} \end{array}$$

where μ is the decay factor of the momentum term, while $g_{t}$ is the accumulated gradient at the *t*-th iteration.

**Diverse Inputs Iterative Fast Gradient Sign Method for Generating Fooling Examples (DI^2^-FGSM^fool^).** DI^2^-FGSM [20] is developed for generating transferable adversarial examples by creating diverse input patterns. DI^2^-FGSM can also be extended into a method for generating transferable fooling examples, which is referred to as DI^2^-FGSM^fool^ and can be expressed as follows:

(4) $$\begin{array}{cc} \; & x_{0}^{fool}=x^{init}\\ \; & x_{t+1}^{fool}={\mathrm{Clip}}_{x}^{\epsilon }\left\{x_{t}^{fool}-\alpha \cdot \mathrm{sign}\left(\nabla_{x}J\left(T\left(x_{t}^{fool};p\right),y\right)\right)\right\} \end{array}$$

where $T(x_{t}^{fool};p)$ indicates a random transformation on the input $x_{t}^{fool}$ with a probability *p*. This transformation may take the form of, e.g., random resizing and padding.

**Translation-Invariant Iterative Fast Gradient Sign Method for Generating Fooling Examples (TI^2^-FGSM^fool^).** Done et al. [21] proposed a translation-invariant method, named TI^2^-FGSM, to generate more transferable adversarial examples against the defense models. TI^2^-FGSM can also be extended into a method for generating transferable fooling examples, which is termed as TI^2^-FGSM^fool^ and can be expressed as follows:

(5) $$\begin{array}{cc} \; & x_{0}^{fool}=x^{init}\\ \; & x_{t+1}^{fool}={\mathrm{Clip}}_{x}^{\epsilon }\left\{x_{t}^{fool}-\alpha \cdot \mathrm{sign}\left(W*\nabla_{x}J\left(x_{t}^{fool},y\right)\right)\right\} \end{array}$$

where W denotes a predefined kernel, which can be uniform, linear, or Gaussian.

### 4.3. Generating Algorithm

The above I-FGSM^fool^, MI-FGSM^fool^, DI^2^-FGSM^fool^, and TI^2^-FGSM^fool^ can be integrated together to form a powerful method, which is referred to as MTI-DI^2^-FGSM^fool^ and presented in Algorithm 1. MTI-DI^2^-FGSM^fool^ is built on the basis of I-FGSM^fool^ by taking advantage of the momentum, diverse inputs, and translation invariance. On the one hand, MTI-DI^2^-FGSM^fool^ is able to achieve high success rates under white-box scenarios; on the other hand, MTI-DI^2^-FGSM^fool^ can effectively improve the transferability of fooling examples under black-box scenarios.
**Algorithm 1 **MTI-DI^2^-FGSM^fool^ for generating fooling examples.**Input**: A classifier *f*; a class label *y*; adversary’s loss function *J*; number of iterations *T*; step size α; perturbation threshold ϵ; decay factor μ; transformation probability *p* and predefined kernel W.**Output**: A fooling example $x^{fool}$ with label *y*.  1:Set $x^{init}$.                    ⊳ random-noise, all-white or all-black.  2:Let $x_{0}^{fool}=x^{init}$, $g_{0}=0$.  3:**for **t=0→T−1** do**  4: Transform $x_{t}^{fool}$ and get $T(x_{t}^{fool};p)$;                    ⊳ Apply DI  5: Input $T(x_{t}^{fool};p)$ to *f* and calculate the gradient: $g_{t}=\nabla_{x}J\left(T\left(x_{t}^{fool};p\right),y\right)$;  6: $g_{t}=W*g_{t}$;                            ⊳ Apply TI  7: $g_{t+1}=\mu \cdot g_{t}+{\frac{g_{t}}{∥g_{t}∥}}_{1}$;                        ⊳ Apply MI  8: $x_{t+1}^{fool}={\mathrm{Clip}}_{x}^{\epsilon }\left\{x_{t}^{fool}-\alpha \cdot \mathrm{sign}\left(g_{t+1}\right)\right\}$;                                 ⊳ Apply FGSM  9:**end for**10:**return **$x_{T}^{fool}$.

## 5. Experiments

### 5.1. Experimental Setup

**Dataset.** Our goal is to generate fooling examples that can be recognized by neural networks. These fooling examples are crafted based on the initial examples by using the algorithm MTI-DI^2^-FGSM^fool^. In our experiments, we consider four types of initial examples: random Gaussian noise images, random uniform noise images, all-white images, and all-black images. Additionally, we need to set the labels that neural networks will recognize the fooling examples as. These labels are taken from an ImageNet-compatible dataset at https://www.kaggle.com/competitions/nips-2017-targeted-adversarial-attack/data (accessed on 16 March 2023), which contains 1000 target labels.

**Models.** We consider four pretrained ImageNet models: ResNet-50 (Res50) [1], Inception-v3 (Inc-v3) [26], DenseNet-121 (Dense121) [27], and VGG16 [28]. These models have diverse architectures and are all publicly available at https://keras.io/api/applications/ (accessed on 16 March 2023).

**Parameter settings and the method for comparison.** Following the settings outlined in [29], for MTI-DI^2^-FGSM^fool^, the number of iterations *T* is set to be 300 and the step size α is set to be 2; the decay factor μ is set to be 1.0; the transformation probability *p* is set to be 0.7; finally, the kernel W is set to be a Gaussian kernel with size of 5. To provide an objective evaluation of our proposed MTI-DI^2^-FGSM^fool^, we compare it with the gradient ascent method [15].

**White-box and black-box scenarios.** In the white-box scenario, we have full access to the model’s architecture and parameters, thus we can use this information to generate fooling examples by directly calculating the gradient of the loss function with respect to the input. In the black-box scenario, we have no direct access to the target model’s architecture or parameters. We may only have access to the model’s input and output. In this case, we can generate fooling examples on one or multiple source models, and then directly feed them to the target model. By leveraging the transferability of fooling examples, the target model may recognize them as the specified objects.

### 5.2. Effect of Loss Function

**Figure 5:**
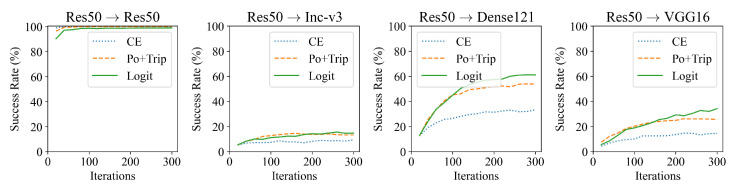
Success rates (%) of fooling examples against four models with different loss functions. The fooling examples are generated on Res50. The initial examples are set to random Gaussian noise images, and ϵ is set to 32.

As for generating adversarial examples, Cross-Entropy (CE) [30] is the most commonly adopted loss function for many attacks. To improve the transferability of the generated adversarial examples, several different loss functions have been developed, such as Po+Trip [31] and logit [29]. We also tested the effect of different loss functions (CE, Po+Trip, and logit) on the success rate of fooling example generation. In more detail, the initial examples are all set to random Gaussian noise images. The maximum threshold ϵ is set to 32. Res50 is used as the source model for fooling example generation. The generated fooling examples are then input into different models and the success rates are observed. The results are presented in Figure 5. As the results show, in the white-box scenario (i.e., on Res50), all three loss functions perform well in generating fooling examples, with success rates close to 100%. However, in the black-box scenario (i.e., on Inc-v3, Dense121, and VGG16), the Logit loss outperforms CE and To+Trip. It is therefore clear that use of the Logit loss produces higher-quality fooling examples; accordingly, Logit loss is used as the default loss function in the following experiments.

### 5.3. Effect of Threshold ϵ

**Figure 6:**
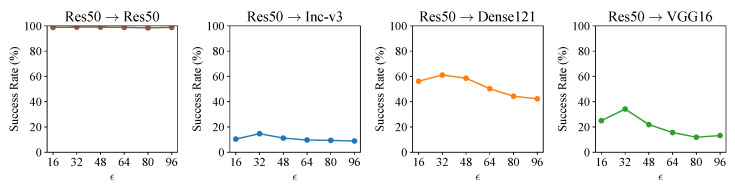
Success rates (%) of fooling examples against four models with different ϵ. The fooling examples are generated on Res50. The initial examples are set to random Gaussian noise images, and the loss function employed is Logit loss.

In the generation of perturbation-based adversarial examples, the parameter ϵ serves as the maximum threshold for restricting the distance between adversarial examples and original benign examples. In our fooling example generation methods, the parameter ϵ is used to restrict the distance between the generated fooling examples and the original initial examples, which guarantees that, like the initial examples, the fooling examples cannot be recognized by humans. We tested the effect of threshold ϵ on the success rate of fooling examples. The results are shown in Figure 6. As we can observe, in the white-box scenario (i.e., on Res50), the value of ϵ has little effect on the success rate of fooling examples, which remains consistently close to 100%. In the black-box scenario (i.e., on Inc-v3, Dense121 and VGG16), the success rates tend to decrease when ϵ>32. We accordingly conclude that the optimal value of ϵ is 32; as such, the parameter ϵ takes the value of 32 by default in the following experiments.

### 5.4. Taking a Look at Fooling Examples

**Figure 7:**
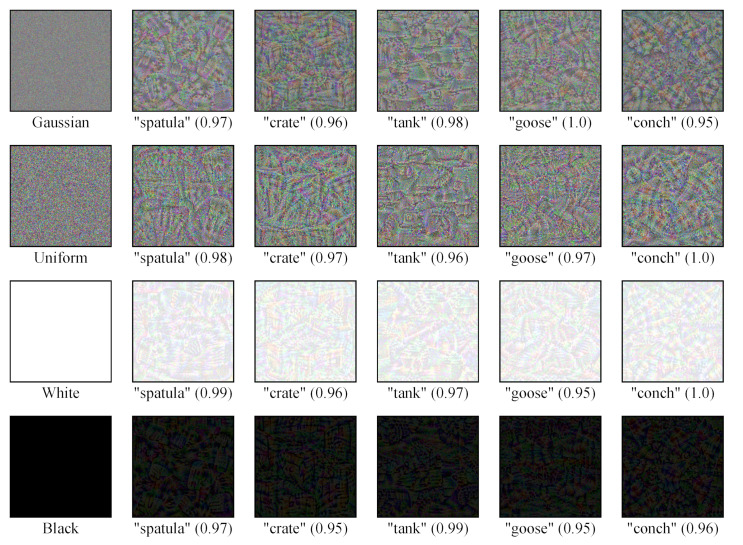
Visualization of fooling examples generated from different initial examples. The fooling examples are generated and tested on Res50. In the above, “‘spatula’ (0.97)” indicates that the fooling example is recognized as “spatula” by Res50 with the confidence of 0.97.

We will now take a look at the fooling examples. Figure 7 visualizes some randomly selected fooling examples generated from different initial examples. As we can observe, all these fooling examples are recognized as natural objects by Res50 with very high confidence. However, we humans are completely unable to recognize these fooling examples.

### 5.5. Generating on a Single Model

We next opt to conduct more extensive experiments. First, we fully test the generation of fooling examples on a single model. Specifically, one model (selected from Res50, Inc-v3, Dense121, and VGG16) is used as the source model to generate fooling examples, after which these generated fooling examples are then tested on all models. The success rates are shown in Table 1; here, the rows represent the source models, and the columns represent the target models. When the source model and target model are the same, it indicates a white-box scenario, in which the generation of fooling examples utilizes the source model’s architecture and parameters; when the source model and target model are different, it indicates a black-box scenario, in which the fooling examples generated on the source model are transferred (i.e., directly input) to the target model. From Table 1, we can observe the following: (a) In the white-box scenario, compared to the gradient ascent method [15], our proposed method has a slightly lower success rate in generating fooling examples in some cases. However, the overall success rate of generating fooling examples in the white-box scenario remains very high, nearly reaching 100%, and is almost unaffected by the settings of initial examples; (b) In the black-box scenario, compared to the gradient ascent method [15], our method achieves a significantly higher success rate in generating fooling examples that are recognized by the target model as the specific objects. We can also observe that the transfer rate of fooling examples is related to the settings of initial examples. Notably, the transfer rate of fooling examples generated from the random Gaussian noise and all-black images is significantly higher than that of fooling examples generated from the random uniform noise and all-white images; (c) The fooling examples generated on Inc-v3 are difficult to transfer to other models (i.e., Res50, Dense121 and VGG16), while the fooling examples generated on other models are also difficult to transfer to Inc-v3.

### 5.6. Generating on an Ensemble of Models

In this section, we test the generation of fooling examples on an ensemble of models. In these experiments, we opt to integrate any three of the four models (i.e., Res50, Inc-v3, Dense121, and VGG16) in order to create an ensemble model for generating fooling examples in the white-box scenario, while the remaining (hold-out) model is used as the black-box model to test the fooling examples. We follow the ensemble strategy proposed in [19] to generate fooling examples on multiple models simultaneously. The success rates are presented in Table 2. From the table, we can observe that compared to the gradient ascent method [15], our method has a slight decrease in the success rate of generating fooling examples on an ensemble model in the white-box scenario. However, in the black-box scenario, our method demonstrates a significant improvement in transferability of fooling examples. We can also observe that compared to the fooling examples generated on a single model, those generated on an ensemble model are significantly more transferable. For MTI-DI^2^-FGSM^fool^, the transfer rate of fooling examples generated from all-black images is the highest, with an average of 64.8%. Contrarily, the transfer rate of fooling examples generated from the random uniform noise images is the lowest, with an average of 8.2%. We can accordingly conclude that the transfer rate of fooling examples is closely related to both the form of source models and the settings of initial examples.

### 5.7. Fooling Google Cloud Vision API

We further test the fooling examples on a real-world computer vision system—Google Cloud Vison API [32]. The Google Cloud Vision API encapsulates powerful machine learning models in an easy-to-use API and is able to quickly classify images into thousands of categories (e.g., “tower”, “lion”, and “sailboat”). We generate fooling examples on an ensemble of four diverse models (i.e., Res50, Inc-v3, Dense121 and VGG 16). The generating algorithm is MTI-DI^2^-FGSM^fool^. The maximum perturbation threshold ϵ is set to 32 and the number of iterations *T* is set to 300. The loss function *J* takes the form of logit loss. The generated fooling examples are sent to the API to observe whether the API classifies the fooling exmaples as the target labels. It is worth noting that for an input image, the API returns up to 10 labels (with confidence ≥50%), and the labels returned by the API are not exactly the same as the labels of the ImageNet models. Therefore, as long as the labels returned by the API contain one label that is semantically similar to the target label, we consider the fooling example to be successfully recognized by the API.

**Figure 8:**
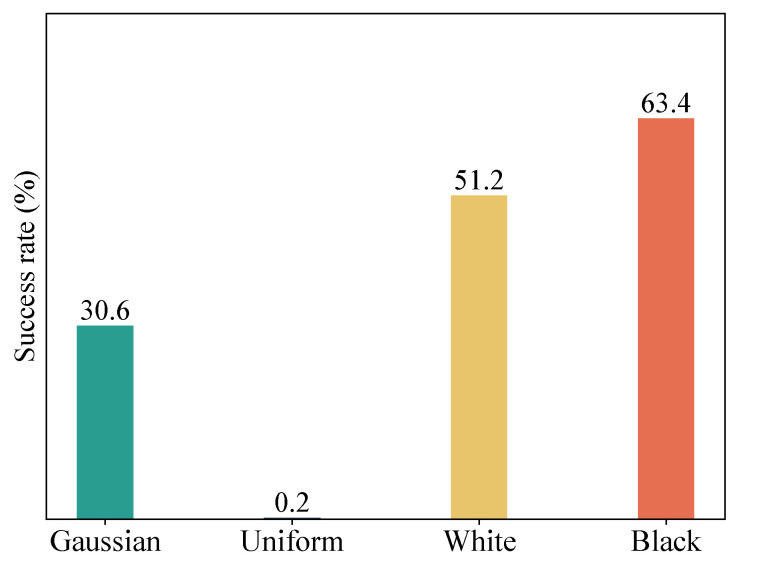
Success rates (%) of recognition of fooling examples by the Google Cloud Vision API.

Figure 8 reports the success rates of recognition of fooling examples by the API. As we can observe, the success rate of fooling examples generated from all-black initial images is the highest (63.4%), followed by fooling examples generated from all-white initial images, with a success rate of 51.2%. The success rate of fooling examples generated from random uniform noise images is the lowest, at only 0.2%.

**Figure 9:**
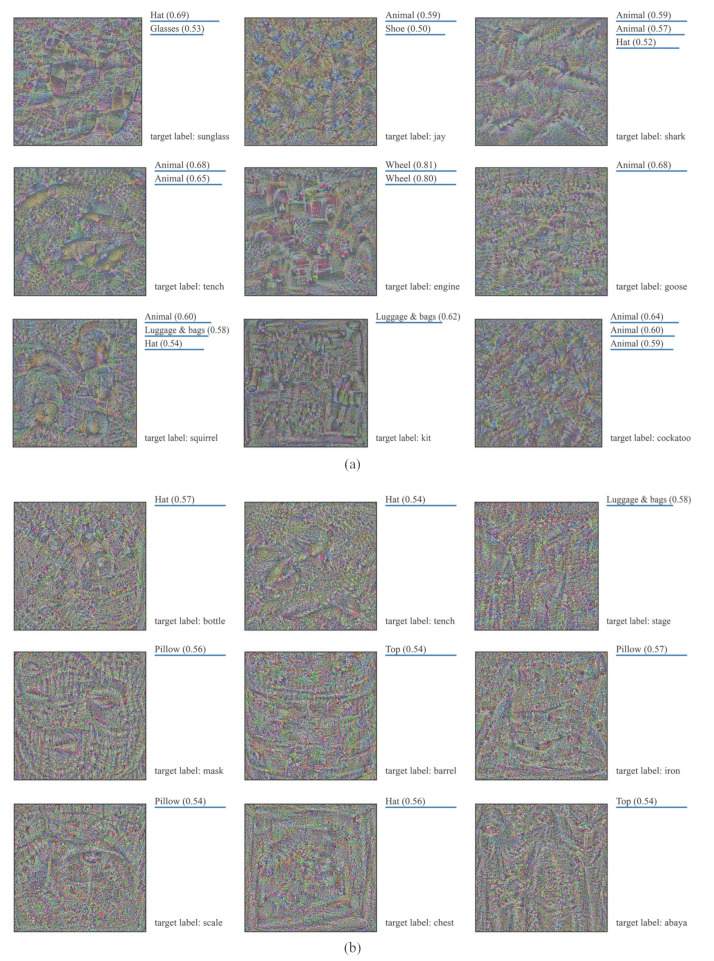
Some fooling examples and the corresponding recognition results of the API: (**a**) fooling examples generated from random Gaussian noise images; (**b**) fooling examples generated from random uniform noise images.

Figure 9 shows some fooling examples generated from random Gaussian or uniform noise images, as well as the API recognition results. It can be seen that the fooling examples generated from random Gaussian noise images are usually recognized by the API as categories that are semantically similar to the target labels, for example, a fooling example with the target label “shark” was recognized as an “animal” by the API. For the fooling examples generated from random uniform noise images, the API hardly recognizes them as target labels.

**Figure 10:**
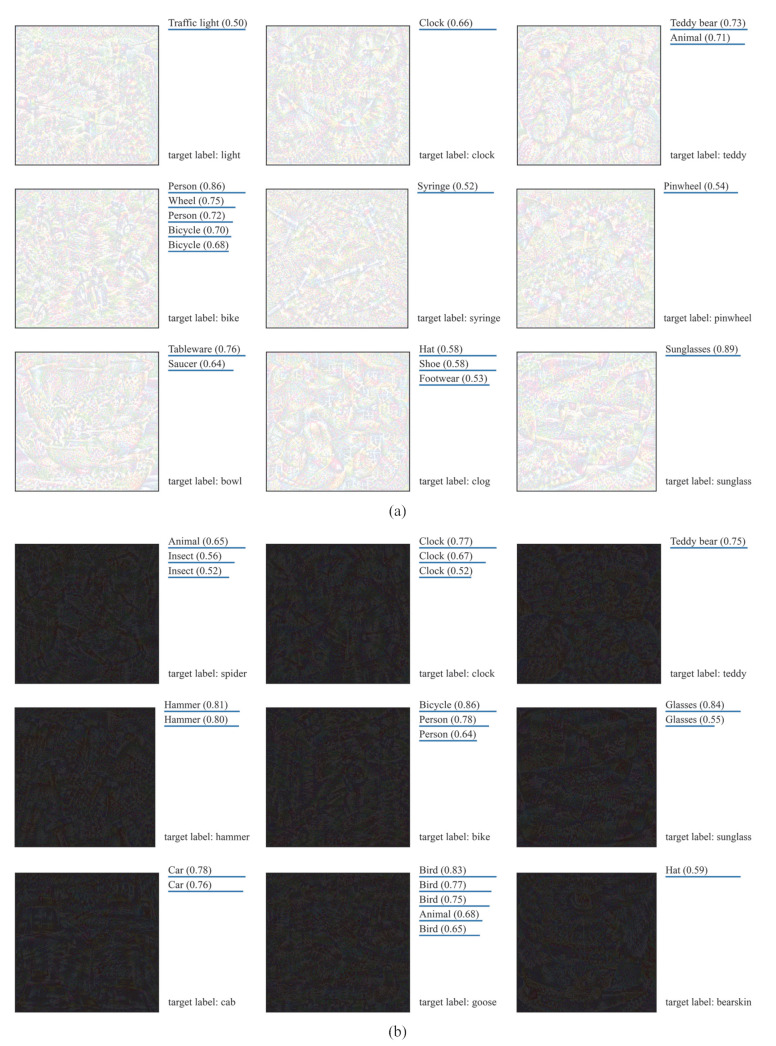
Some fooling examples and the corresponding recognition results of the API: (**a**) fooling examples generated from all-white initial images; (**b**) fooling examples generated from all-black initial images.

Figure 10 shows some fooling examples generated from all-white or all-black initial images, as well as the API recognition results. It can be seen that the success rate of the API correctly recognizing these fooling examples is quite high, and these fooling examples can make the API accurately recognize them as the target labels. For example, a fooling examples generated from an all-white initial image with the target label “teddy” was recognized by the API as “Teddy bear” with a confidence of 0.73; a fooling example generated from an all-black initial image with the target label “hammer” was recognized by the API as “Hammer” with a confidence of 0.81.

## 6. Conclusions

In this paper, we propose the concept of fooling examples, which refer to examples that can be recognized by neural networks but not by humans. We first discuss the differences between fooling and adversarial examples. Next, we develop an iterative method for generating fooling examples, which combines techniques such as momentum, diverse inputs, and translation invariance to improve the transferability of fooling examples. Finally, we conduct extensive experiments on well-trained ImageNet models to generate fooling examples. The results show that fooling examples can not only be easily generated in the white-box scenario, but also exhibit strong transferability across different models in the black-box scenario.

## Figures and Tables

**Table 1:** Success rates (%) of fooling examples generated on a single model. The blocks with a white background indicate white-box scenarios, while the blocks with a gray background indicate black-box scenarios. Bolded numbers indicate superior results.

Model	Initial Example	Method	Res50	Inc-v3	Dense121	VGG16
Res50	Gaussian noise image	Gradient ascent [15]	98.8	1.3	3.1	0.8
		MTI-DI${}^{2}$-FGSM${}^{\mathrm{fool}}$ (Ours)	**99.0**	**14.7**	**61.1**	**34.2**
	Uniform noise image	Gradient ascent [15]	**99.1**	0.2	0.3	0.4
		MTI-DI${}^{2}$-FGSM${}^{\mathrm{fool}}$ (Ours)	98.7	**2.7**	**20.8**	**1.8**
	All-white image	Gradient ascent [15]	**99.0**	0.7	1.9	3.2
		MTI-DI${}^{2}$-FGSM${}^{\mathrm{fool}}$ (Ours)	95.9	**5.1**	**27.6**	**37.5**
	All-black image	Gradient ascent [15]	99.0	0.8	24.6	17.0
		MTI-DI${}^{2}$-FGSM${}^{\mathrm{fool}}$ (Ours)	**99.0**	**8.9**	**66.5**	**50.7**
Inc-v3	Gaussian noise image	Gradient ascent [15]	**0.9**	**99.3**	0.5	0.7
		MTI-DI${}^{2}$-FGSM${}^{\mathrm{fool}}$ (Ours)	0.6	98.9	**3.0**	**1.6**
	Uniform noise image	Gradient ascent [15]	0.4	**99.5**	0.0	0.4
		MTI-DI${}^{2}$-FGSM${}^{\mathrm{fool}}$ (Ours)	**0.4**	99.2	**2.1**	**0.7**
	All-white image	Gradient ascent [15]	0.5	**97.8**	1.1	1.0
		MTI-DI${}^{2}$-FGSM${}^{\mathrm{fool}}$ (Ours)	**2.2**	97.3	**1.4**	**1.8**
	All-black image	Gradient ascent [15]	0.9	99.1	1.5	1.2
		MTI-DI${}^{2}$-FGSM${}^{\mathrm{fool}}$ (Ours)	**4.5**	**99.4**	**3.1**	**2.5**
Dense121	Gaussian noise image	Gradient ascent [15]	2.1	2.3	**99.1**	2.1
		MTI-DI${}^{2}$-FGSM${}^{\mathrm{fool}}$ (Ours)	**10.5**	**8.7**	98.8	**18.0**
	Uniform noise image	Gradient ascent [15]	0.5	0.1	**98.5**	0.4
		MTI-DI${}^{2}$-FGSM${}^{\mathrm{fool}}$ (Ours)	**3.8**	**3.8**	98.3	**1.5**
	All-white image	Gradient ascent [15]	2.5	1.1	**98.0**	5.2
		MTI-DI${}^{2}$-FGSM${}^{\mathrm{fool}}$ (Ours)	**10.3**	**4.0**	97.3	**13.4**
	All-black image	Gradient ascent [15]	9.5	1.9	**99.0**	10.5
		MTI-DI${}^{2}$-FGSM${}^{\mathrm{fool}}$ (Ours)	**34.5**	**7.0**	98.4	**16.0**
VGG16	Gaussian noise image	Gradient ascent [15]	0.2	0.6	0.6	**96.7**
		MTI-DI${}^{2}$-FGSM${}^{\mathrm{fool}}$ (Ours)	**1.1**	**1.4**	**10.3**	96.3
	Uniform noise image	Gradient ascent [15]	0.4	0.3	0.5	**96.3**
		MTI-DI${}^{2}$-FGSM${}^{\mathrm{fool}}$ (Ours)	**1.3**	**0.9**	**2.1**	96.0
	All-white image	Gradient ascent [15]	0.9	0.5	1.2	94.0
		MTI-DI${}^{2}$-FGSM${}^{\mathrm{fool}}$ (Ours)	**4.2**	**0.6**	**3.7**	**94.3**
	All-black image	Gradient-based [15]	3.2	1.2	6.1	94.3
		MTI-DI${}^{2}$-FGSM${}^{\mathrm{fool}}$ (Ours)	**20.7**	**1.7**	**20.9**	**95.0**

**Table 2:** Success rates (%) of fooling examples generated on an ensemble of models. Here, “-Res50” indicates that the fooling examples are generated on an ensemble model of Inc-v3, Dense121 and VGG16, and the generated fooling examples are tested on Res50 (i.e., the hold-out model). The meaning of other symbols can be deduced by analogy. Bolded numbers indicate superior results.

Model	Initial Example	Method	-Res50	-Inc-v3	-Dense121	-VGG16
Ensemble	Gaussian noise image	Gradient ascent [15]	**99.7**	**99.7**	**99.9**	**99.5**
		MTI-DI${}^{2}$-FGSM${}^{\mathrm{fool}}$ (Ours)	99.6	99.2	99.4	99.3
	Uniform noise image	Gradient ascent [15]	**99.9**	**99.7**	**99.9**	**99.7**
		MTI-DI${}^{2}$-FGSM${}^{\mathrm{fool}}$ (Ours)	99.3	99.4	99.5	99.6
	All-white image	Gradient ascent [15]	98.9	98.4	98.5	**99.7**
		MTI-DI${}^{2}$-FGSM${}^{\mathrm{fool}}$ (Ours)	**99.3**	**98.6**	**99.2**	99.1
	All-black image	Gradient ascent [15]	99.1	99.0	99.1	**99.8**
		MTI-DI${}^{2}$-FGSM${}^{\mathrm{fool}}$ (Ours)	**99.7**	**99.2**	**99.5**	99.7
Hold-out	Gaussian noise image	Gradient ascent [15]	2.1	3.1	4.3	2.0
		MTI-DI${}^{2}$-FGSM${}^{\mathrm{fool}}$ (Ours)	**26.5**	**42.4**	**68.2**	**52.8**
	Uniform noise image	Gradient ascent [15]	0.5	0.4	0.7	0.4
		MTI-DI${}^{2}$-FGSM${}^{\mathrm{fool}}$ (Ours)	**5.2**	**8.6**	**16.6**	**2.3**
	All-white image	Gradient ascent [15]	4.7	3.9	5.0	17.1
		MTI-DI${}^{2}$-FGSM${}^{\mathrm{fool}}$ (Ours)	**37.8**	**19.3**	**60.5**	**56.0**
	All-black image	Gradient ascent [15]	37.4	9.6	55.4	53.5
		MTI-DI${}^{2}$-FGSM${}^{\mathrm{fool}}$ (Ours)	**72.2**	**31.4**	**84.3**	**71.3**

## Data Availability

The data presented in this study are openly available at https://github.com/mingcheung/fooling-examples (accessed on 25 April 2023).
